# The Role of Active or Passive Drainage after Evacuation of Chronic Subdural Hematoma: An Analysis of Two Randomized Controlled Trials (cSDH-Drain-Trial and TOSCAN Trial)

**DOI:** 10.3390/diagnostics12123045

**Published:** 2022-12-05

**Authors:** Florian Ebel, Ladina Greuter, Katharina Lutz, Levin Häni, Javier Fandino, Raphael Guzman, Luigi Mariani, Jürgen Beck, Andreas Raabe, Werner J. Z’Graggen, Philippe Schucht, Jehuda Soleman

**Affiliations:** 1 Department of Neurosurgery, University Hospital of Basel, 4031 Basel, Switzerland; 2 Department of Neurosurgery, Inselspital, University Hospital of Bern, 3010 Bern, Switzerland; 3 Department of Neurosurgery, Klinik Hirslanden, 8032 Zürich, Switzerland; 4 Faculty of Medicine, University of Basel, 4031 Basel, Switzerland; 5 Department of Neurology, Inselspital, University Hospital of Bern, 3010 Bern, Switzerland; 6 Clinical Trial Unit, University Hospital of Basel, 4031 Basel, Switzerland

**Keywords:** chronic subdural hematoma, drainage, burr hole trepanation, recurrence

## Abstract

The evacuation of a chronic subdural hematoma (cSDH) is one of the most common procedures in neurosurgery. The aim of this study was to assess the influence of drainage suction in the surgical treatment of cSDH on the recurrence rate. Post hoc analysis was conducted on two randomized controlled trials (cSDH-Drain-Trial and TOSCAN trial) stratifying a total of 581 patients into active or passive drain type. Of the 581 patients, 359 (61.8%) and 220 (37.9%) were stratified into the active and passive drainage groups, respectively. The reoperation rate following postoperative recurrence was 23.1% and 14.1% in the active and passive drainage groups, respectively (*p* < 0.011). After propensity score matching, the differences in recurrence rate remained significant (26.6% versus 15.6%, *p* = 0.012). However, the functional outcome (mRS) at 6–12 months did not differ significantly (median [IQR]) between the 2 groups (passive drainage group 0.00 [0.00, 2.00], active drainage group 1.00 [0.00, 2.00], *p* = 0.431). Mortality was comparable between the groups (passive drainage group 12 (5.5%), active drainage group 20 (5.6%), *p* = 0.968). In the univariate analysis, active drainage, short (<48 h) duration of drainage, and early (<48 h) postoperative mobilization were significantly associated with a higher recurrence rate. However, the multivariate logistic regression model could not confirm that any of these parameters were significantly associated with recurrence. Our post hoc analysis proposes that using a passive instead of an active drain might be associated with a reduced recurrence rate after evacuation of a cSDH. We suggest gathering further evidence by means of a randomized controlled trial.

## 1. Introduction

Chronic subdural hematoma (cSDH) is one of the most common pathologies treated by neurosurgery [1]. It occurs mainly in elderly patients, and with the aging of the population, an increase in its incidence is expected [2]. The gold standard treatment for cSDH is surgical evacuation through burr-hole trepanation (BHT), with the insertion of a drain [3]. The recurrence rate of surgically treated cSDH is approximately 10% [4]. There is good evidence for the benefit of drain insertion after BHT, and placement of a subperiosteal drain, rather than a subdural drain, is recommended because although the recurrence rate is similar, it has a lower complication rate [4,5]. However, opinions are divided on further technical aspects, such as the application of suction (active drainage), irrigation, duration of drainage, postoperative positioning, and postoperative mobilization [6,7,8,9,10,11,12,13]. The literature concerning the application of suction or mobilization, and whether these factors influence the recurrence rate or outcome after cSDH, is scarce [6,7,8].

The aim of this post hoc analysis was to compare the recurrence rate and clinical outcomes between active and passive drainage following BHT for cSDH.

## 2. Materials and Methods

### 2.1. Patient Population

**Figure 1:**
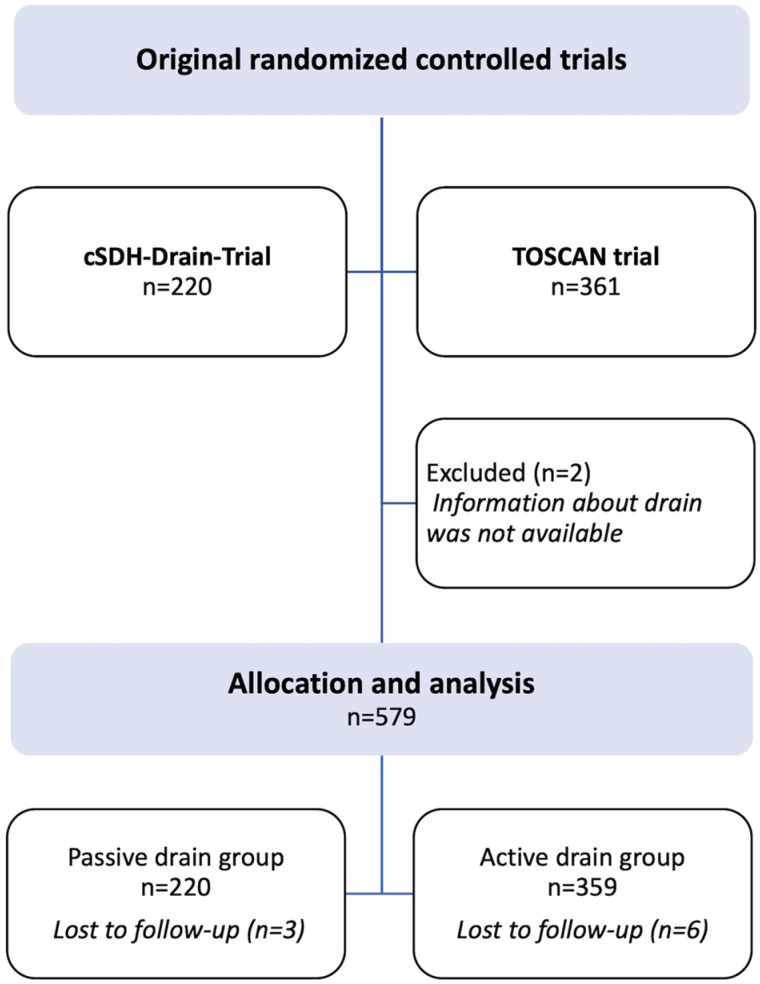
Flowchart displaying inclusion and group assignment of patients.

This is a post hoc analysis of the influence of an active or passive drain on the recurrence rate after BHT based on the data from earlier randomized controlled trials (RCT), the cSDH-Drain-Trial and the TOSCAN trial [5,14,15]. The cSDH-Drain-Trial randomized participants to receive either a subperiosteal or subdural drain and compared the recurrence rate after BHT. The TOSCAN trial randomized participants to receive either clinical or radiological follow-up with computed tomography (CT) on days 2 and 30 after BHT and assessed favorable clinical outcomes at 6 months. In both studies, the secondary outcome parameters were clinical and radiological outcome measures, morbidity, and mortality, whereas in the TOSCAN trial, recurrence was analyzed secondarily as well. During the TOSCAN trial, the institutional guidelines were changed to allow only subperiosteal drains instead of subdural drains. Therefore, of the 359 patients in the TOSCAN trial included in this post hoc analysis, 135 received a subdural and 214 a subperiosteal drain. A total of 579 patients—cSDH-Drain-Trial (220); TOSCAN trial (359)—were analyzed, after 2 patients were excluded due to missing drain information (Figure 1). The 2 RCTs had a high rate of short and long term follow-up, with very few patients lost to follow-up (TOSCAN trial 6 (1.7%), cSDH-Drain-Trial 3 (1.4%)) [5,15] (Figure 1).

Demographic data on the preoperative clinical condition (age, sex, comorbidities, Glasgow Coma Scale (GCS), and mRS), as well as radiological parameters (midline shift (MLS) and diameter of hemorrhage) were assessed. The original trials each had different follow-up time points, which we combined for this analysis into follow-up at 24–48 h, 4–6 weeks, and 6–12 months. Radiologic outcomes at 24–48 h and 4–6 weeks included residual hematoma diameter and residual midline shift. Postoperative CT results were unavailable for those patients in the TOSCAN trial who were not randomized to the CT group, unless they had presented postoperatively with clinical worsening or a new neurological deficit. Common medical complications assessed were deep vein thrombosis, pulmonary embolism, and pneumonia. We assessed surgery-related complications, such as the different types of postoperative bleeding (epidural hematoma and intracerebral hemorrhage), as well as surgical infections. In the TOSCAN trial, postoperative bleeding into the subdural space was considered a recurrence, whether it was acute or chronic. In the cSDH-Drain-Trial, postoperative acute bleeding into the subdural space was counted as a hemorrhage complication in terms of acute SDH. We adjusted for this dissimilarity and ultimately defined a recurrence as radiological evidence of a hematoma, either an acute SDH or cSDH, on the ipsilateral side with clinical symptoms requiring surgery within 6 (TOSCAN trial) or 12 months (cSDH-Drain-Trial). Mortality rates from the cSDH-Drain-Trial were adjusted to 6 months to make this consistent with the TOSCAN trial.

### 2.2. Surgical and Postoperative Management

The surgical technique was very similar in both trials. Evacuation was performed with a double burr-hole trepanation (frontal and parietal), washing out of the hematoma with Ringer’s lactate, and insertion of either a subdural or subperiosteal drain. Surgery was generally performed under general anesthesia. In the cSDH-Drain-Trial, a passive drain was inserted and left in situ for 48 h (passive drain group; PDG) in all patients, whereas in the TOSCAN trial, suction was applied to the drain (active drain group; ADG), and the drain remained in situ for 24–48 h. For passive drainage, a Jackson–Pratt drain was used without applying negative pressure to the drain bulb. For active drainage, a Jackson–Pratt drain, with the application of negative pressure by compressing the drain bulb, was used. In both trials, a single-shot antibiotic (cefuroxime) was administered 30–45 min before surgery. In the TOSCAN trial, an additional 1.5 g of cefuroxime was administered every 8 h until the drain was removed. None of the patients in the cSDH-Drain-Trial were mobilized and remained in a flat position whenever possible for the first 48 h after surgery. Patients in the TOSCAN trial were mobilized individually during the first 48 h postoperatively (bed rest with upper body flat (2.3%), bed rest with upper body at 30° (15.3%), partial mobilization (33.9%), free mobilization (47.1%)).

### 2.3. Primary and Secondary Endpoints

This study compared the recurrence rate as the primary outcome, while secondary outcome measures were the time to recurrence, the clinical outcome measured by mRS and GCS, and the radiological parameters such as MLS and postoperative surgical and medical complications, as mentioned above. Additionally, univariate and logistic regression analysis was carried out to identify possible risk factors for recurrence. The clinical outcome score was trichotomized (mRS: good = 1–3, bad = 4–5, dead = 6; and GCS: good = 14–15, moderate = 9–13, bad = 3–8).

### 2.4. Statistical Analysis

Statistical analysis was performed using R statistical software (Vienna, Austria, version 4.0.3). The Mann–Whitney U test was used for continuous variables, and analyses were presented as the median and interquartile range. The interquartile range is presented in square brackets throughout the text and tables. Either the chi-square test or Fisher exact test, depending on the number of variables, was used for categorical data, and the results are presented as number of participants (%). Multivariate logistic regression (categorical variables) or Poisson regression (continuous variables) analysis was carried out for significant variables in the univariate analysis to compare the association of different suction regimens with the outcome parameters. Time to recurrence in the active and passive drain groups was compared by the log-rank test and presented as a Kaplan–Meier curve. As the 2 groups showed differences in some of their baseline characteristics, we conducted a 1:1 propensity score matching for age and GCS at admission to reduce this bias. After the propensity matching, a total of 384 patients were matched and analyzed according to their baseline characteristics and outcome parameters (Supplementary Tables S1 and S2). A *p*-value of <0.05 was considered significant.

## 3. Results

### 3.1. Baseline Characteristics

Of the 581 study participants recruited between June 2012 and August 2016, 220 (37.9%) were included in the passive drain group (PDG) and 359 (61.8%) in the active drain group (ADG). The mean age was 74.90 (±10.85) years. Age, GCS, and mRS at presentation were significantly different between the groups (Table 1). A subperiosteal drain was inserted in 334 (57.7%) of patients and was equally distributed among the groups (PDG 120 (54.5%) and ADG 214 (59.6%), *p* = 0.131). Nearly half of the patients presented with headaches (289 (49.9%)) and more than a third with motor deficits (213 (36.8%)), with a significant difference between the groups (Table 1).

### 3.2. Primary and Secondary Outcomes

**Figure 2:**
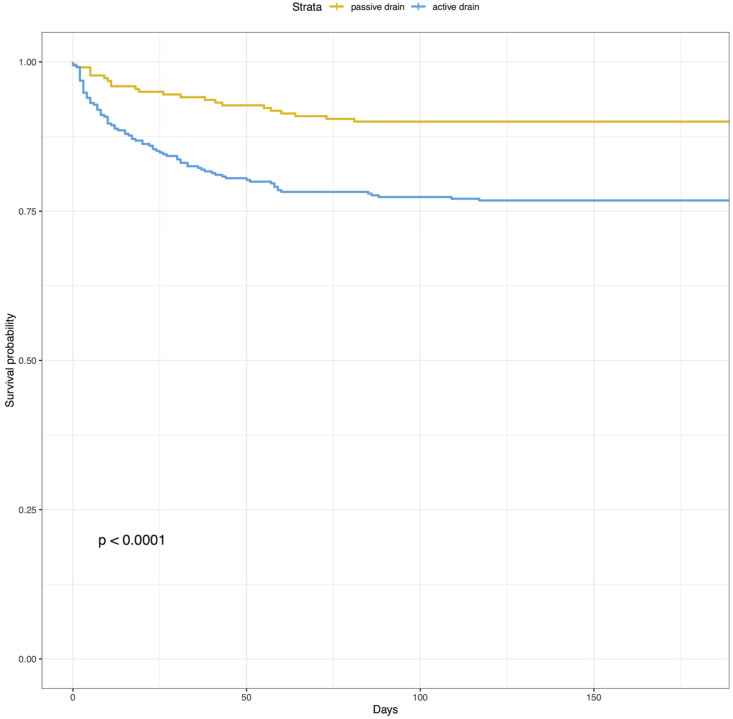
Kaplan–Meier curve of time to recurrence stratified by the drainage groups.

**Figure 3:**
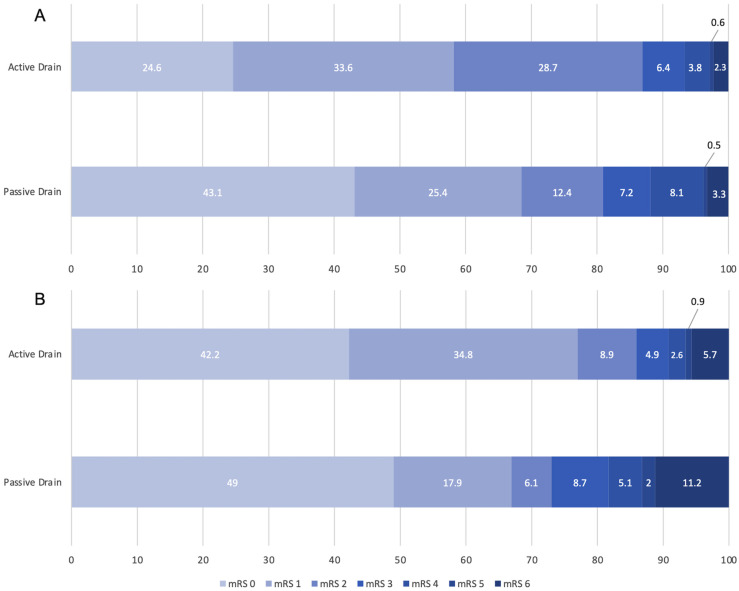
Stacked bar charts showing the functional outcome modified Rankin Scale (**A**) at 4–6 weeks and (**B**) 6–12 months postoperatively, stratified by active and passive drainage.

Patients in the passive drain group showed a significantly lower recurrence rate (14.1% (n = 31)) compared to patients in the active drain group (23.1% (n = 83), *p* < 0.011). Time to recurrence was significantly longer in the passive drain group (chi-square, *p* = 16.0 and log-rank test x^2^ *p* < 0.001; Figure 2). Median [IQR] postoperative GCS at 24–48 h and 4–6 weeks were significantly lower in the passive drain group (15.00 [14.00, 15.00] versus 15.00 [15.00, 15.00], *p* < 0.001 and 15.00 [15.00, 15.00] versus 15.00 [15.00, 15.00], *p* < 0.001). However, both groups had a similar functional outcome at 4–6 weeks follow-up (mRS in the PDG 1.00 [0.00, 2.00] and ADG 1.00 [0.50, 2.00], *p* = 0.261, Table 2, Figure 3A).

At 6–12 months, patients in the passive drain group had a significantly worse mRS (1.00 [0.00, 3.00] versus 1.00 [0.00, 1.00], *p* = 0.018, Table 2, Figure 3B) and a lower GCS (15.00 [15.00, 15.00] and 15.00 [15.00, 15.00], *p* = 0.019, Table 2) compared to those in the active drain group. After propensity score matching for age and GCS at admission, the significant difference in recurrence rate between the active and passive drain groups remained (PDG 30 (15.6%) and ADG 51 (26.6%), *p* = 0.012), while no significant difference was observed in outcome at 6–12 months (mRS in the PDG 0.00 [0.00, 2.00] and ADG 1.00 [0.00, 2.00], *p* = 0.431, Supplementary Table S2).

Radiological outcome parameters showed similar mean MLS 24–48 h postoperatively (PDG 0.34 (±0.28) cm and ADG 0.34 (±0.32) cm, *p* = 0.893), but a significantly smaller mean MLS was observed in the passive drain group at follow-up after 4–6 weeks postoperatively (PDG 0.09 (±0.19) cm and ADG 0.16 (±0.31) cm, *p* = 0.005, Table 2). Surgical and medical complications showed no statistically significant difference between the passive and active drain groups (Table 2). Surgical infections were significantly higher in the passive than in the active drain group (PDG 11 (5.0%)) and ADG 6 (1.7%), *p* = 0.047). The mortality rates were comparable between the active and passive drain groups (ADG 20 (5.6%) and PDG 12 (5.5%), *p* = 0.968).

### 3.3. Risk Factors for Recurrence

Univariate analysis of the whole cohort found suction, drain duration, and type of mobilization in the first 48 h to be significantly associated with recurrence (Table 3). However, the multivariate logistic regression model did not confirm that any of the parameters mentioned above were significantly associated with recurrence (Table 4).

## 4. Discussion

Our study showed a significantly lower recurrence rate of cSDH after BHT associated with insertion of a passive (14.1%) compared to an active drain (23.1%). Furthermore, in the univariate analysis, perioperative factors such as early drain removal or early mobilization of the patient after surgery were associated with higher recurrence rates.

Due to the rising incidence and prevalence of cSDH, it is critical not only for the patient’s benefit, but also for ensuring cost efficiency, to standardize surgical treatment and postoperative management to minimize the risk of recurrence and optimize the clinical outcome. Surprisingly, although BHT is one of the most common neurosurgical procedures, there is significant variability in its execution [16]. Only the benefits of the insertion of a drain are widely recognized and supported by strong evidence [4,16]. Similarly, there is strong evidence showing that insertion of a subperiosteal drain leads to similar recurrence rates and fewer complications compared to subdural drain [5,17]. To date, no consensus exists on whether active or passive drainage should be used, on the length of time for which the drain should be left in situ, or on the ideal timing of postoperative mobilization after BHT [16]. Furthermore, the timely diagnosis of recurrence is important. However, as the TOSCAN study showed, regular postoperative CT imaging has no benefit compared to clinical follow-up alone [15]. In the presence of clinical deterioration or persisting neurologic deficits, the diagnostic modality of choice is a native CT imaging of the skull to detect a possible recurrence of cSDH [18].

In Austria, Germany, and Switzerland, after BHT in cSDH, passive drains are inserted significantly more frequently (72.2%) than active drains (23.6%) [16]. Several case series describe experiences with different types of drains and drainage techniques (active, passive, or continuous irrigation drains) [6,7]. However, data enabling a direct comparison between active and passive drains and their impact on the recurrence of cSDH are scarce. The largest study, by Sjavik et al., retrospectively analyzed 1260 patients to investigate the effect of different drainage types, such as active subperiosteal, passive subdural, and continuous irrigation drains, on recurrence rates. Overall, patients with a passive subdural drain required reoperation due to recurrence significantly more often (20%) than patients with a continuous irrigation drain (10.8%), or an active subperiosteal drain (11.1%) [8]. However, continuous subdural irrigation drains were significantly more often associated with postoperative complications than the other 2 types of drainage [8]. This would suggest that a passive drain is associated with a higher risk of recurrence. However, Sjavik et al. did not compare passive versus active drains alone, but rather passive subdural drains with irrigation compared to active subperiosteal drains. As shown in previous trials, subdural drains are associated with a higher complication rate than subperiosteal drains. Moreover, it is not clear whether the higher reoperation or complication rate is due to the type of drainage, subdural placement, or to continuous irrigation. In contrast, our data showed a significantly lower recurrence rate in patients with passive (14.1%) compared to active drains (23.1%). These significant differences persisted, even after propensity score matching (Supplementary Table S2). The functional outcome at 6–12 months postoperatively was initially shown to be worse in patients with passive drainage. However, after propensity score matching, the significant difference was no longer evident, indicating that the previously observed difference is probably due to differences in the demographic characteristics of the participants and not to the type of drainage used (Table 2, Supplementary Table S2). The incidence of postoperative bleeding, such as epidural hematoma or intracerebral hemorrhage, was similar in both groups. However, it should be emphasized that 50% of the TOSCAN trial patients did not receive a postoperative CT scan, whereas all patients in the cSDH-Drain-Trial did, and thus some postoperative asymptomatic bleeding may not have been diagnosed.

Furthermore, our data showed that postoperative infections were significantly more frequent in the passive compared to the active drain group (5% versus 1.7%, *p* = 0.047). Compared to patients with passive drains, patients with active drains received postoperative antibiotics every 8 h until the drain was removed. There is, however, no solid evidence that prolonged administration of prophylactic systemic antibiotics in patients with drains in place resulted in a reduced rate of surgical site infections [19]. A meta-analysis of 7 published RCTs that assessed prolonged prophylactic use of systemic antibiotics versus perioperative prophylaxis alone in patients with surgical drains failed to show significant differences in the incidence of surgical site infection [20].

Interestingly, in our work, as in the study by Sjavik et al., there was a clear difference in the recurrence rate between the different cohorts and the different perioperative management of cSDH. We suspect that apart from the drain’s suction effect, other variables related to perioperative management, such as drain duration and postoperative mobilization, could play an essential role. So far, few studies have investigated the effect of drain duration postoperatively on recurrence rates and outcome, and no consensus has yet been reached. It is common to remove the drain 48 h postoperatively, but results in the literature are controversial [16,21,22]. A study by Glancz et al. found no difference in recurrence rate whether the drain was removed on the first or second postoperative day [23]. However, Kale et al., using retrospectively collected data, showed that the recurrence rate in patients with a drain in situ for 2–4 days postoperatively was significantly higher than in patients who had the drain for 5–7 days (15.6% versus 3.3%). In a recently randomized controlled trial, it was shown that there was no difference in recurrence rate between a drain duration of 24 or 48 h, respectively [24]. The univariate analysis of our data showed a significant association between short drain duration and recurrence (Table 3). However, in the multivariate logistic regression, drain duration was not shown to be a significant predisposing factor for recurrence (Table 4). Further interventional trials are certainly needed to elucidate this matter.

Postoperative mobilization after the evacuation of a cSDH is an essential aspect, as the patients are often elderly and may have several comorbidities. Therefore, these patients are prone to complications such as deep vein thrombosis, pulmonary embolism, or pneumonia when immobilized [25]. There are no guidelines or consensus on the timing for postoperative mobilization. According to an international survey by Baschera et al., 66% of neurosurgeons completely abstained from prescribing postoperative bed rest, whereas 16% and 12%, respectively, prescribed bed rest for 24 h and 24–48 h postoperatively [16]. To better distinguish between the effects of the timing of mobilization and the type of drainage, we additionally analyzed the recurrence rate between the active and passive drainage group only in patients prescribed bedrest for 48 h. This analysis still found a significantly higher recurrence rate in the active drainage group (Supplementary Table S3). This might indicate that regardless of postoperative mobilization, active drainage is associated with higher recurrence rates. According to the literature, delayed postoperative mobilization is generally associated with a significantly worse functional outcome and a higher risk for postoperative complications [9,21]. On the other hand, specifically in patients treated for cSDH, postoperative bed rest in the supine position could support brain re-expansion and thus, may reduce the risk of recurrence [25]. Two prospective randomized controlled studies investigated the effect of postoperative posture on recurrence risk after BHT, with controversial results [10,11].

In our univariate analysis, we identified early mobilization as a predictive factor for recurrence, while medical complication rate, including thromboembolic events, was comparable in patients who were mobilized late (results not shown).

More interventional studies are needed to acquire high-quality data for further analysis and risk stratification.

## 5. Limitations

This study has several limitations. First, in this analysis, findings from two randomized controlled trials conducted at different centers were pooled. Even though the study protocols were comparable, there were likely regional differences in treatment indications, follow-up routines, or indications for reoperations that may have affected our findings. Using propensity score matching, we attempted to minimize these biases. Furthermore, in the TOSCAN trial, only 50% of the patients received a routine postoperative CT. This may have resulted in underdiagnosis of clinically silent postoperative bleeding complications. Additionally, in each study, some parameters were missing, and some patients were lost to follow-up, which might reduce the validity of the results, despite the low rate of missing data. Finally, no systematic search for thromboembolic events was performed in either study, which reduces the power of the analysis of postoperative mobilization with respect to thromboembolic complications.

## 6. Conclusions

Based on our post hoc analysis, the insertion of a passive drain after BHT of cSDH might be associated with lower recurrence rates, comparable outcome rates, and higher infection rates. Furthermore, a short duration of postoperative drainage (<48 h) and early postoperative mobilization (within 48 h) seem to be associated with an increased risk of recurrence. Due to possible confounding factors, causality is not assured. To provide a clear recommendation regarding active or passive drainage, an RCT is recommended.

## Figures and Tables

**Table 1:** Baseline characteristics of both groups.

Drain Group	Passive	Active	*p*-Value
n	220	359	
Sex = male (%)	149 (67.7)	243 (67.7)	1
Age (mean ±SD)	77.25 (10.06)	73.46 (11.07)	<0.001
Drain type (%)			0.13
subdural	100 (45.5)	135 (37.6)	
subperiosteal	120 (54.5)	214 (59.6)	
Mobilization for 48 h (%)			<0.001
Bed rest, flat	220 (100.0)	8 (2.3)	
Bed rest, 30°	0 (0.0)	54 (15.3)	
Partial mobilization	0 (0.0)	122 (33.9)	
Full mobilization	0 (0.0)	169 (47.1)	
Drain duration (mean ±SD), hours	48.00 (0.00)	34.60 (11.58)	<0.001
GCS at presentation (median [IQR])	15.00 [14.00, 15.00]	15.00 [15.00, 15.00]	<0.001
GCS at presentation grouped (%)			<0.001
good	179 (81.4)	333 (92.8)	
moderate	35 (15.9)	24 (6.7)	
bad	6 (2.7)	2 (0.6)	
mRS at presentation (median [IQR])	2.00 [1.00, 3.00]	1.00 [1.00, 2.00]	<0.001
mRS at presentation grouped = good (%)	169 (76.8)	334 (93.0)	<0.001
MLS preoperative (mean ±SD), cm	0.73 (0.52)	0.74 (0.50)	0.871
Aphasia = yes (%)	47 (21.4)	80 (22.3)	0.876
Motor deficit = yes (%)	108 (49.1)	105 (29.2)	<0.001
Headache = yes (%)	67 (30.6)	222 (61.8)	<0.001
Urinary incontinence = yes (%)	6 (2.7)	10 (2.8)	1
Seizure = yes (%)	12 (5.5)	13 (3.6)	0.399
Hypertension = yes (%)	30 (13.6)	57 (15.9)	0.54
Diabetes mellitus = yes (%)	30 (13.6)	54 (15.5)	0.631
CAD = yes (%)	63 (28.6)	98 (27.3)	0.8
Atrial fibrillation = yes (%)	46 (20.9)	91 (25.3)	0.263
Stroke = yes (%)	27 (12.3)	26 (7.2)	0.059
COPD = yes (%)	5 (2.3)	12 (3.3)	0.627
Smoking = yes (%)	8 (3.6)	18 (5.0)	0.569
Alcohol = yes (%)	13 (5.9)	13 (3.6)	0.279
DVT = yes (%)	8 (3.6)	21 (6.0)	0.288
Blood thinner = yes (%)	133 (60.5)	191 (53.2)	0.105

CAD = coronary artery disease, COPD = chronic obstructive pulmonary disease, DVT = deep venous thrombosis, GCS = Glasgow Coma Scale, mRS = modified Rankin Scale, MLS = midline shift, SD = standard deviation.

**Table 2:** Outcome parameters of both groups.

Drain Group	Passive	Active	*p*-Value
n	220	359	
Recurrence = yes (%)	31 (14.1)	83 (23.1)	**0.011**
Time to recurrence (mean ±SD), days	21.16 (24.99)	23.41 (25.58)	0.228
GCS after 24–48 h (median [IQR])	15.00 [14.00, 15.00]	15.00 [15.00, 15.00]	**<0.001**
GCS grouped after 24–48 h (%)			**<0.001**
good	195 (88.6)	357 (99.7)	
moderate	22 (10.0)	1 (0.3)	
bad	3 (1.4)	0 (0.0)	
GCS after 4–6 weeks (median [IQR])	15.00 [15.00, 15.00]	15.00 [15.00, 15.00]	**<0.001**
GCS grouped after 4–6 weeks (%)			0.256
good	197 (97.5)	327 (99.7)	
moderate	4 (2.0)	1 (0.3)	
bad	1 (0.5)	0 (0)	
mRS after 4–6 weeks (median [IQR])	1.00 [0.00, 2.00]	1.00 [0.50, 2.00]	0.261
mRS grouped after 4–6 weeks (%)			0.087
good	184 (88.0)	322 (93.3)	
bad	18 (8.6)	15 (4.3)	
GCS after 6–12 months (median [IQR])	15.00 [15.00, 15.00]	15.00 [15.00, 15.00]	0.019
GCS grouped after 6–12 months			0.256
good	171 (98.3)	316 (99.7)	
moderate	3 (1.7)	1 (0.3)	
mRS after 6–12 months (median [IQR])	1.00 [0.00, 3.00]	1.00 [0.00, 1.00]	0.018
mRS grouped after 6–12 months (%)			0.008
good	160 (81.6)	316 (90.8)	
bad	14 (7.1)	12 (3.4)	
Diameter of remaining hematoma after 24–48 h (mean ±SD) cm	1.05 (0.54)	1.17 (0.56)	0.029
Diameter of remaining hematoma after 4–6 weeks (mean ±SD), cm	0.56 (0.57)	0.72 (0.59)	0.007
MLS 24–48 h postoperative (mean ±SD), cm	0.34 (0.28)	0.34 (0.32)	0.893
MLS 4–6 weeks postoperative (mean ±SD), cm	0.09 (0.19)	0.16 (0.31)	0.005
Hemorrhagic complications = yes (%)	5 (2.3)	6 (1.7)	0.877
Hemorrhage type (%)			0.424
EDH	1 (0.5)	0 (0.0)	
ICB	4 (1.8)	6 (1.7)	
Surgical infections = yes (%)	11 (5)	6 (1.7)	0.047
Medical complication (%)			0.440
DVT	2 (0.9)	2 (0.6)	
PE	2 (0.9)	2 (0.6)	
Pneumonia	3 (1.4)	1 (0.3)	
Mortality = yes (%)	12 (5.5)	20 (5.6)	0.968
Mortality, surgery related = yes (%)	5 (2.3)	0 (0.0)	0.073

ASDH = acute subdural hematoma, cm = centimeter, CSDH = chronic subdural hematoma, DVT = deep vein thrombosis, EDH = epidural hematoma, GCS = Glasgow Coma Scale, ICB = intracerebral bleed, M = months, MLS = midline shift, mRS = modified Rankin Scale, PE = pulmonary embolism, W = weeks.

**Table 3:** Univariate analysis of potential risk factors for recurrence.

Recurrence	No	Yes	*p*-Value
n	465	114	
Suction = yes (%)	276 (59.4)	83 (72.8)	0.011
Sex = male (%)	311 (66.9)	81 (71.1)	0.458
Age (mean ±SD), years	74.80 (10.83)	75.29 (10.96)	0.669
Drain type (%)			0.998
subdural	189 (40.6)	46 (40.4)	
subperiosteal	268 (57.6)	66 (57.9)	
both	8 (1.7)	2 (1.8)	
Mobilization for 48 h (%)			0.043
Bed rest, flat	194 (42.1)	34 (30.4)	
Bed rest, 30°	37 (8.0)	17 (15.2)	
Partial mobilization	100 (21.5)	22 (19.3)	
Full mobilization	130 (28)	39 (34.2)	
Drain duration (mean ±SD), hours	43.53 (9.10)	40.34 (11.42)	0.013
Blood thinner = yes (%)	253 (54.4)	71 (62.3)	0.158
MLS preoperative (mean ±SD), cm	0.72 (0.47)	0.78 (0.62)	0.256
Hematoma width, preoperative (mean (±SD)), cm	1.96 (0.76)	2.06 (0.74)	0.207
Membranes n (mean ±SD)	0.88 (0.94)	0.75 (0.95)	0.188
Motor deficit, preoperative = yes (%)	179 (38.5)	34 (29.8)	0.107
Hypertension = yes (%)	75 (16.1)	12 (10.5)	0.176
CAD = yes (%)	121 (26.0)	40 (35.1)	0.069
Atrial fibrillation = yes (%)	110 (23.7)	27 (23.7)	1
COPD = yes (%)	12 (2.6)	5 (4.4)	0.475
Dementia = yes (%)	29 (6.2)	7 (6.1)	1
Smoking = yes (%)	21 (4.5)	5 (4.4)	1
Alcohol = yes (%)	22 (4.7)	4 (3.5)	0.755
GCS at presentation (median [IQR])	15.00 [14.00, 15.00]	15.00 [14.00, 15.00])	0.246
GCS at presentation grouped (%)			0.571
good	408 (87.7)	104 (91.2)	
moderate	50 (10.8)	9 (7.9)	
bad	7 (1.5)	1 (0.9)	
mRS at presentation (median [IQR])	2.00 [1.00, 3.00]	2.00 [1.00, 2.00]	0.352
mRS at presentation grouped = good (%)	399 (85.8)	104 (91.2)	0.167

CAD = coronary artery disease, COPD = chronic obstructive pulmonary disease, cm = centimeter, GCS = Glasgow Coma Scale, MLS = midline shift, mRS = modified Rankin Scale.

**Table 4:** Multivariate logistic regression model for potential risk factors identified in the univariate analysis.

Recurrence	Odds Ratio (OR)	95% Confidence Interval	*p*-Value
Suction = yes (%)			
no	Ref.		
yes	2.4	0.1–30.5	0.51
Drain duration (mean ±SD), hours	0.99	0.95–1.03	0.67
Mobilization within the first 48 h			
Bed rest, flat	Ref.		
Bed rest, 30°	1.1	0.09–24.8	0.96
Partial mobilization	0.06	0.002–2.1	0.08
Full mobilization	0.99	0.09–22.4	0.99

## Data Availability

The data presented in this study are available on request from the corresponding author.

## Supplementary Materials

The Supplementary Tables S1–S3 can be accessed and downloaded at https://www.mdpi.com/article/10.3390/diagnostics12123045/s1, Table S1: Baseline characteristics of both groups after propensity score matching for age and GCS at presentation; Table S2: Outcome parameters of both groups after propensity score matching; Table S3: Outcome parameters of both groups after excluding patients not on bed rest.
