# Supplementary material for: The Role of Active or Passive Drainage after Evacuation of Chronic Subdural Hematoma: An Analysis of Two Randomized Controlled Trials (cSDH-Drain-Trial and TOSCAN Trial)
Source: Diagnostics (Basel). 2022 Dec 5;12(12):3045. doi: 10.3390/diagnostics12123045 (PMC9777194; doi:10.3390/diagnostics12123045)
Supplement: Supplementary file 1 [file diagnostics-12-03045-s001.zip › diagnostics-2001152-supplementary.pdf]

Supplementary Table S1: **Baseline characteristics of both groups after propensity score matching for age and GCS at presentation**

**Drain group**	**Passive**	**Active**	**p-value**
n	192	192	
Age (mean (SD))	76.70 (9.67)	76.02 (10.29)	0.5
Sex = male (%)	131 (68.2)	119 (62.0)	0.239
Drain type = subperiosteal (%)	104 (54.2)	102 (53.1)	0.918
Drain duration (mean (±SD)), hours	48.00 (0.00)	34.85 (11.47)	**<0.001**
Mobilization for 48 hours (%)			**<0.001**
Bed rest, flat	192 (100.0)	4 (2.1)	
Bed rest, 30°	0 (0.0)	38 (20.3)	
Partial mobilization	0 (0.0)	66 (34.4)	
Full mobilization	0 (0.0)	89 (46.4)	
GCS at presentation (median [IQR])	15.00 [14.00, 15.00]	15.00 [14.00, 15.00]	0.152
mRS at presentation (median [IQR])	2.00 [1.00, 3.00]	2.00 [1.00, 2.00]	**0.007**
Aphasia = yes (%)	38 (19.8)	51 (26.6)	0.147
Motor deficit = yes (%)	92 (47.9)	62 (32.3)	**0.003**
Headache = yes (%)	63 (33.0)	111 (57.8)	**<0.001**
Incontinence = yes (%)	4 (2.1)	6 (3.1)	0.749
Seizure = yes (%)	12 (6.2)	7 (3.6)	0.347
Hypertension = yes (%)	27 (14.1)	29 (15.1)	0.885
CAD = yes (%)	54 (28.1)	56 (29.2)	0.91
Atrial fibrillation = yes (%)	42 (21.9)	53 (27.6)	0.237
Stroke = yes (%)	24 (12.5)	19 (9.9)	0.517
COPD = yes (%)	5 (2.6)	6 (3.1)	1
Smoker = yes (%)	8 (4.2)	8 (4.2)	1
Alcohol = yes (%)	12 (6.2)	4 (2.1)	0.074
CAD = yes (%)	54 (28.1)	56 (29.2)	0.91
Blood thinner = yes (%)	115 (59.9)	111 (57.8)	0.756
MLS preoperative cm (mean (±SD))	0.72 (0.51)	0.75 (0.52)	0.509

mRS = modified Rankin Scale, MLS = midline shift, CAD = coronary artery disease, COPD = chronic obstructive pulmonary disease, GCS = Glasgow Coma Scale

Supplementary Table S2: **Outcome parameters of both groups after propensity score matching**

**Drain group**	**Passive**	**Active**	**p-value**
n	192	192	
Recurrence = yes (%)	30 (15.6)	51 (26.6)	**0.012**
Time to 1st recurrence (days) (mean (±SD))	3.43 (12.82)	6.40 (17.17)	0.056
Mortality = yes (%)	8 (4.3)	15 (7.8)	0.844
GCS after 24–48 hours (median [IQR])	15.00 [14.00, 15.00]	15.00 [15.00, 15.00]	**<0.001**
GCS after 4–6 weeks (median [IQR])	15.00 [15.00, 15.00]	15.00 [15.00, 15.00]	**<0.001**
GCS after 6–12 months (median [IQR])	15.00 [15.00, 15.00]	15.00 [15.00, 15.00]	0.21
mRS after 4–6 weeks (median [IQR])	1.00 [0.00, 1.75]	1.00 [1.00, 2.00]	**0.002**
mRS after 6–12 months (median [IQR])	0.00 [0.00, 2.00]	1.00 [0.00, 2.00]	0.431
MLS 24–48 hours postoperative (mean (±SD)), cm	0.34 (0.27)	0.34 (0.34)	0.832
MLS 4–6 weeks postoperative (mean (±SD)), cm	0.08 (0.18)	0.16 (0.27)	**0.006**
Diameter of remaining hematoma after 24–48 hours (mean (±SD)), cm	1.05 (0.54)	1.24 (0.59)	**0.007**
Diameter of remaining hematoma after 4–6 weeks (mean (±SD)), cm	0.55 (0.55)	0.72 (0.60)	**0.020**
Hemorrhagic complications = yes (%)	4 (2.1)	5 (2.6)	1
Hemorrhage type (%)			0.472
EDH	1 (0.5)	0 (0.0)	
ICB	3 (1.6)	5 (2.6)	
Surgical Infection = yes (%)	9 (4.7)	3 (1.6)	0.143
Medical complication (%)			0.143
Deep vein thrombosis	2 (1.0)	1 (0.5)	
Pulmonary embolism	2 (1.0)	0 (0.0)	
Pneumonia	3 (1.6)	0 (0.0)	

mRS = modified Rankin Scale, MLS = midline shift, cm = centimeter, W = weeks, M = months, EDH = Epidural hematoma, ICB = Intracerebral bleed, CSDH = chronic subdural hematoma, GCS = Glasgow Coma Scale

Supplementary Table S3: **Outcome parameters of both groups after excluding patients not on bed rest**

**Drain group**	**Passive**	**Active**	**p-value**
n	220	60	
Recurrence = yes (%)	31 (14.1)	19 (31.7)	**0.004**
Time to 1st recurrence, days (mean (±SD))	3.01 (12.02)	9.52 (20.54)	**0.002**
Mortality = yes (%)	12 (5.7)	5 (8.3)	0.709
GCS after 24–48 hours (median [IQR])	15.00 [14.00, 15.00]	15.00 [15.00, 15.00]	**<0.001**
GCS after 4–6 weeks (median [IQR])	15.00 [15.00, 15.00]	15.00 [15.00, 15.00]	**0.002**
GCS after 6–12 months (median [IQR])	15.00 [15.00, 15.00]	15.00 [15.00, 15.00]	**0.017**
mRS after 4–6 weeks (median [IQR])	1.00 [0.00, 2.00]	1.00 [0.00, 2.00]	0.184
mRS after 6–12 months (median [IQR])	1.00 [0.00, 3.00]	1.00 [0.00, 2.00]	0.659
MLS 24–48 hours postoperative (mean (±SD)), cm	0.34 (0.28)	0.30 (0.35)	0.457
MLS 4–6 weeks postoperative (mean (±SD)), cm	0.09 (0.19)	0.18 (0.23)	**0.022**
Diameter of remaining hematoma after 24–48 hours (mean (±SD)) cm	1.05 (0.54)	1.15 (0.51)	0.336
Diameter of remaining hematoma after 4–6 weeks (mean (±SD)), cm	0.56 (0.57)	0.73 (0.64)	0.121
Hemorrhagic complications = yes (%)	4 (1.8)	1 (1.7)	1
Type of hemorrhage, postoperative (%)			0.859
EDH	1 (0.5)	0 (0.0)	
ICB	3 (1.4)	1 (1.7)	
Surgical Infection = yes (%)	11 (5.0)	2 (3.3)	0.843
Medical complication (%)			0.347
Deep vein thrombosis	2 (0.9)	2 (3.3)	
Pulmonary embolism	2 (0.9)	0 (0.0)	
Pneumonia	3 (1.64)	0 (0.0)	

mRS = modified Rankin Scale, MLS = midline shift, cm = centimeter, W = weeks, M = months, EDH = Epidural hematoma, ICB= Intracerebral bleed, CSDH = chronic subdural hematoma, GCS = Glasgow Coma Scale
